# Association of extended myositis panel results, clinical features, and diagnoses: a single-center retrospective observational study

**DOI:** 10.1007/s00296-021-05012-0

**Published:** 2021-10-04

**Authors:** Shamma Ahmad Al Nokhatha, Eman Alfares, Luke Corcoran, Niall Conlon, Richard Conway

**Affiliations:** 1 Department of Rheumatology, St. James’s Hospital, James’s Street, Dublin, Ireland; 2 Department of Immunology, St. James’s Hospital, James’s Street, Dublin, Ireland

**Keywords:** Myositis, Autoimmune, Antibodies, Inflammatory

## Abstract

Myositis-specific antibodies (MSA) and myositis-associated antibodies (MAA) are a feature of the idiopathic inflammatory myopathies (IIM), but are also seen in other rheumatic diseases, and in individuals with no clinical symptoms. The aim of this study was to assess the clinical utility of MSA and MAA and in particular the clinical relevance of weakly positive results. We included all patients at our institution who had at least one positive result on the Immunoblot EUROLINE myositis panel over a 6-year period (2015–2020). Associations with clinical features and final diagnosis were evaluated. Eighty-seven of 225 (39%) myositis panel tests met the inclusion criteria. There were 52 strong positives and 35 weak positives for one or more MSA/MAAs. Among the strong positive group, 15% (8/52) were diagnosed with IIM, 34.6% (18/52) with interstitial lung disease, 7.7% (4/52) with anti-synthetase syndrome, 25% (13/52) with connective tissue disease, and others accounted for 25% (13/52). In weak-positive cases, only 14% (5/35) had connective tissue disease and none had IIM. 60% (21/35) of weak-positive cases were not associated with a specific rheumatic disease. A significant number of positive myositis panel results, particularly weak positives, are not associated with IIM or CTD.

**Supplementary Information:**

The online version contains supplementary material available at 10.1007/s00296-021-05012-0.

## Introduction

The idiopathic inflammatory myopathies (IIM) are a heterogeneous group of autoimmune rheumatic diseases characterized by proximal muscle weakness and frequent involvement of other organ systems [1]. The prevalence of IIM can be estimated between 2.4 and 33.8 per 100,000 persons [2].

Historically, the Bohan and Peter criteria were used for IIM, until 2017 when the European League Against Rheumatism and American College of Rheumatology (EULAR/ACR) proposed new classification criteria [3, 4]. These new classification criteria reflect the advances of medicine in the last 40 years as well as providing higher performance (sensitivity/specificity, 93%/88% with biopsies, 87%/82% without biopsies). The new criteria are based primarily on clinical history, examination, and biopsy results. Only one antibody, Anti-Jo-1, is included. The criteria are in the form of a calculator which gives a probability score of the patient having myositis. A classification tree is then used to help determine the subcategory (polymyositis (PM), dermatomyositis (DM), inclusion body myositis, and juvenile dermatomyositis) [4].

However, autoantibodies have been reported in more than 80% of patients with IIM. These autoantibodies can be myositis-specific antibodies (MSA), or myositis-associated antibodies (MAA) which are also seen in a host of other connective tissue diseases (CTD). MSA have a 90% diagnostic specificity, while MAA are noted in up to 50% of myositis patients. These antibodies can help anticipate the clinical course and disease prognosis [5, 6].

MSA include anti-ARS (aminoacyl-tRNA synthetases) antibodies; (histidyl (Jo-1), threonyl (PL-7), alanyl (PL-12), glycyl (EJ), isoleucyl (OJ), asparaginyl (KS), tyrosyl (Ha), and phenylalanyl (Zo)), anti-Mi2 (nucleosome-remodeling deacetylase complex), anti-SRP (signal recognition particle), anti-TIF1 (transcription intermediary factor 1) and anti-NXP-2 (nuclear matrix protein 2), anti-MDA5 (melanoma differentiation-associated protein 5), and anti-SAE (small ubiquitin-like modifier activating enzyme). MAA include anti-PM-Scl, U1RNP, Ku, and Ro52 [7–9].

Autoantibodies are a feature of the subclinical phase of systemic rheumatic diseases and can be present for many years before the onset of clinical symptoms [10, 11]. MSA and MAA are associated with IIM; however, only anti Jo-1 is included in the EULAR/ACR criteria. Weak-positive MSA/MAA are frequently seen and of uncertain clinical significance. Therefore, the aim of the study is to assess the clinical utility of MSA and MAA and in particular the clinical relevance of weakly positive results.

## Materials and methods

### Study design and setting

This study is a single-center retrospective observational study, performed over a 6-year period (2015–2020). All patients who had an extended myositis antibody panel in this period were assessed for eligibility. Those over age 18 with at least one positive MSA/MAA were included and patients who were followed up in other institutions were excluded. IIM patients with positive MSA/MAA were compared to weak-positive MSA/MAA patients. The study was approved by the St. James’ Hospital (SJH)/Tallaght University Hospital (TUH) Joint Research Ethics Committee under protocol number 2020–04 List 15, in May 2020.

### Determination/procedure

Myositis antibody testing was performed using the Immunoblot EUROLINE myositis panel, according to the manufacturer’s specifications. This assay allows the detection of human IgG autoantibodies to a range of different antigens. This includes 12 MSA (Mi-2a, Mi-2b, TIF1, MDA5, NXP2, SAE1, SRP, Jo-1, PL-7, PL-12, EJ, and OJ), in addition to 4 MAA (Ku, PM-Scl100, PM-Scl75, and Ro/SSA-52). Our immunology lab reports PM-Scl100 and PM-Scl75 separately. Some consider both anti-PM-Scl100 and anti-PM-Scl75 antibodies as one, since they target two closely related isoforms of the same protein. For the purpose of this study, we have included those who were positive for PM-Scl75 and/or PM-Scl100 under the one result. The same applies for Mi-2a and Mi-2b [12]. Anti-nuclear antibody (ANA) screening by indirect immunofluorescence (IIF) on HEp-2 cells is performed in tandem with each myositis panel to improve specificity, as some myositis antibodies have a distinct ANA staining pattern [13]. The assay was performed according to the manufacturer’s recommendations, using a screening dilution of 1:80. Comments are on the presence or absence of antibodies, in addition to the pattern.

### Measurement

Immunoblot strips were analyzed using the EuroBlotOne Analyzer/Euroline Scan. This assay provides a semi-qualitative result based on signal intensity of each measured antibody. Results are reported as: negative, weak positive, and strong positive. According to the manufacturer’s recommendations, an antibody is considered negative if the signal is < 11. Low positivity is a signal between 11 and 25, and strong positivity beyond 25. The turnover time for the assay is 21 days.

### Clinical features

Clinical features were defined as follows. Interstitial lung disease was diagnosed by a respiratory physician. Other features were identified by a rheumatologist and/or immunologist. Arthritis was defined as swelling and tenderness of one or more joints, arthralgia as joint pain with no evidence of arthritis, myositis as muscle weakness supported by relevant investigations, Raynaud’s phenomenon as recurrent events of sharply demarcated pallor and/or cyanosis of the skin of the digits with or without reactive hyperaemia, and cutaneous manifestations as Gottron’s papules or sign, heliotrope rash, photosensitive rash, calcinosis, digital ulceration, psoriasis, livedo reticularis, or sclerodactyly. Malignancy was defined as any cancer within 5 years of the index study.

### Statistical analysis

Statistical analysis was performed using SPSS v26. Descriptive statistics were reported, with results given as frequency and percentages. Categorical variables were compared using Chi-square tests. *p* ≤ 0.05 was considered statistically significant throughout.

## Results

### Patients and demographics

A total of 225 myositis panels were performed in the 6-year study period. 87/225 (39%) patients had positive myositis panel results and met the inclusion criteria, 39% were male and 61% female, with a mean (SD) age of 58 (+ -16) years. Of the positive results, 60% (52/87) were strong positive for and 40% (35/87) weak positive for one or more MSA/MAAs. Full demographic data are shown seen in Table 1 (strong positive cohort) and Table 2 (weak-positive cohort).

**Table 1:** Strong-positive myositis panel characteristics

	ANA	Age/gender	MAA	MSA	ILD	Arthritis	Arthralgia	Myositis	Raynaud	Cutaneous	Malignancy	Final diagnosis	Treatment	Outcome
Inflammatory myositis														
1	+ S	43M	Ro52			** + **	** + **	** + **		** + **		Dermatomyositis Hidradenitis suppurativa	Prednisolone + HCQ	Remission/stable
2	+ S	76F		NXP2				** + **		** + **		Dermatomyositis Myasthenia gravis	Prednisolone + IVIG + Azathioprine + pyridostigmine	Remission/stable
3	+ S	45F	Ro52					** + **		** + **	** + **	Paraneoplastic dermatomyositis, stage 4 high-grade serous ovarian carcinoma	Prednisolone + MMF + IVIG + chemotherapy	Worsening
4	−	54F		MDA5			** + **			** + **		Amyopathic dermatomyositis	Prednisolone + MTX	Remission/stable
5	+ S	42F		SAE1				** + **		** + **		Dermatomyositis	Prednisolone + MTX	Remission/stable
6	−	77M		Mi2b						** + **		Dermatomyositis	Topical corticosteroid	Remission/stable
7	−	55M	PMscl100/75 Ro52				** + **			** + **		Dermatomyositis	Prednisolone + MTX	Remission/stable
8	+ H	62F	PMscl100/75							** + **		Dermatomyositis sine myositis	MTX	Remission/stable
Interstitial lung disease														
9	−	66F	Ro52	PL12	** + **							IPF	Prednisolone	Died
10	+ S	55M		SAE1/OJ	** + **							IPF	No medication	Lost follow-up
11	+	68M	Ro52		** + **							IPF	Prednisolone	Remission/stable
12	+ S	72F	PMscl100/75		** + **							IPF	Pirfenidone	Remission/stable
13	−	83M		PL12	** + **							IPF	No medication	Remission/stable
14	−	73M		EJ	** + **							IPF	No medication	Remission/stable
15	−	78M	Ro52		** + **							IPF	Pirfenidone	Remission/stable
16	C	46M	Ro52		** + **		** + **			** + **		IPAF Pyoderma gangrenosum	Prednisolone Adalimumab	Remission/stable
17	+ S	53M	Ro52		** + **				** + **			IPAF	Prednisolone + MMF	Remission/stable
18	−	72F		PL12	** + **						** + **	IPAF	Under evaluation	Remission/stable
19	+ H	85F	PMscl100/75		** + **							IPAF	Prednisolone	Remission/stable
20	C	71F	Ro52	PL7	** + **	** + **	** + **		** + **	** + **		Anti-synthetase syndrome	Prednisolone Cyclophosphamide then Azathioprine	Remission/stable
21	C	62M		PL7	** + **			** + **				Anti-synthetase syndrome	Prednisolone + Rituximab	Remission/stable
22	−	43M		JO-1	** + **				** + **			Anti-synthetase syndrome	No medication	Remission/stable
23	−	66F	Ro52	JO-1	** + **							Anti-synthetase syndrome	Prednisolone + MMF then rituximab	Remission/stable
24	−	73M		SAE1/SRP	** + **						** + **	Progressive pulmonary fibrosis (post COVID, ARDS and recurrent aspiration) Esophageal Ca T1N2M0 s/p esophagectomy	Antibiotics + supportive care	Remission/stable
25	C	66M	Ro52		** + **	** + **	** + **					RA-ILD	Prednisolone + Rituximab	Remission/stable
26	−	74F	Ro52		** + **		** + **					Sjogren -ILD	Prednisolone + AZA + HCQ	Remission/stable
Connective tissue disease														
26	+ S	60F	Ro52									SLE	HCQ	Lost follow-up
27	+ H	75F	Ro52			** + **	** + **					Sjogren	HCQ	Remission/stable
28	+ S	69F	Ro52									Sjogren	HCQ	Remission/stable
29	+ S	53F	Ro52				** + **			** + **	** + **	Sjogren Breast cancer	No medication Surgery + Radiotherapy + Hormonal	Remission/stable
30	+ S	18F	Ro52	EJ			** + **					Sjogren	HCQ	Remission/stable
31	−	33M	Ro52				** + **					Sjogren with neuropsychiatry manifestation	AZA	Remission/stable
32	+	73F	Ro52				+					Sjogren	HCQ	Remission/stable
34	+ `	66F	Ku/Ro52				** + **			** + **		Undifferentiated CTD	No medication	Lost follow-up
35	+ S	19 F	U1snRNP			** + **	** + **		** + **			Undifferentiated CTD	Prednisolone MTX + HCQ	Remission/stable
36	−	70M	Ro52						** + **		** + **	Undifferentiated CTD query paraneoplastic on background melanoma and eosinophilia	Nifedipine	Remission/stable
37	+ S	48F	U1snRNP/ Ro52	OJ		** + **	** + **	** + **		** + **		MCTD Autoimmune hepatitis	Prednisolone + AZA + HCQ	Remission/stable
38	+ Ce	52F		SRP					** + **	** + **		Limited cutaneous scleroderma	Nifedipine	Remission/stable
39	+	54F	PMscl100/75						** + **	** + **		Scleroderma Scleroderma renal crisis	HCQ and ramipril	Remission/stable
Others														
40	−	72M		NXP2			** + **					Polymyalgia rheumatica	Prednisolone	Remission/stable
41	+ S	61M	ku	Mi2b			** + **					large vessel vasculitis	Prednisolone Tocilizumab	Remission/stable
42	−	35F		PL12		** + **	** + **			** + **		PsA	MTX	Remission/stable
43	+ S	49F		Mi2b								PBC	Ursodeoxycholic acid	Remission/stable
44	+ N	50F	Ro52									Liver cirrhosis	No medication	Remission/stable
45	+ S	53 F	Ro52									Autoimmune limbic encephalitis	IV methylpred + IVIG + plasma exchange + cyclophosphamide	Died
46	+ N	46F	Ro52				** + **					Fibromyalgia	No medication	Remission/stable
47	−	37F	PMscl100/75 Ro52				** + **					Fibromyalgia	No treatment	Remission/stable
48	C	45F	Ku/Ro52									Chronic spontaneous urticaria Hypothyroidism	Anti-histamine Levothyroxine	Remission/stable
49	−	71F	Ro52								** + **	High grade serous ovarian carcinoma with metastasis	Surgery and chemotherapy	Remission/stable
50	+ H	62F	Ro52									Uterine fibroid	No treatment	Remission/stable
51	+ H	64F	Ro52			** + **						Rheumatoid arthritis	MTX	Remission/stable
52	−	73F	PMscl100/75					** + **				Extranodal NK/T lymphoma	-	Died

*S* speckled, *H* homogenous, *C* cytoplasmic, *Ce* centromere, *N* nucleolar

**Table 2:** Weak-positive myositis panel characteristics

	Age/gender	ANA	MAA	MSA	ILD	Arthritis	Arthralgia	Myositis	Raynaud	Cutaneous	Malignancy	Final diagnosis	Medications	Outcome
1	19M	–	PMscl 100/75	EJ OJ		** + **	** + **					IBD-related spondyloarthropathy	Adalimumab	Remission/stable
2	29F	–		TIF1								MDR TB and neuropathy Intrauterine fibroid	Antibiotic Pregabalin	Remission/stable
3	32M	–		Mi2b						** + **		Psoriasis	No medication	Remission/stable
4	63F	–		Mi2b	** + **							Asymptomatic idiopathic bi-apical fibrosis	No medication	Remission/stable
5	57M	–		SRP	** + **							Sarcoidosis Bilateral interstitial pulmonary fibrosis	Nintedanib	Lost follow-up
6	54M	–		Mi2		** + **	** + **			** + **		Scleroderma, psoriatic arthritis	Prednisolone + MTX	Remission/stable
7	79F	–		SRP	** + **							IPF query RA related	Prednisolone	Died
8	52F	C		Mi2b								Fatty liver along with hepatosplenomegaly Hypothyroidism	No medication	Remission/stable
9	54M	–	Ro52								** + **	NSCLC-adenocarcinoma T2N1M0 + antiphospholipid syndrome and VTE history	Prednisolone + chemotherapy	Remission/stable
10	59F	C	Ro52							** + **		Idiopathic livedo vs erythema ab igne	No medication	Remission/stable
11	71M	+ S	U1snRNP	PL12				** + **				Poorly controlled Myasthenia Gravis Coeliac disease Hypothyroidism	IVIG + steroid + pyridostigmine	Remission/stable
12	43F	+ H	Ro52	PL7		** + **				** + **		UCTD	HCQ + MMF	Remission/stable
13	56M	–	PMscl 100/75 Ro52		** + **		** + **		** + **			Scleroderma/pulmonary fibrosis IgA deficiency	Prednisolone + Rituximab + MTX	Remission/stable
14	73F	–		MDA5/SAE1								Degenerative lumbosacral spine	–	Remission/stable
15	66M	+ H	PmScl 100/75				+					No Unclear diagnosis—paroxysms of inflammation cause unclear	No medications	Remission/stable
16	41F	–	PmScl 100/75						+			Raynaud phenomenon	Supportive	Remission/stable
17	67F	+ H	Ro52									MGUS	–	Remission/stable
18	18F	–		Mi2a/b SRP								Chilblains likely secondary to anorexia nervosa	–	Remission/stable
19	61M	–		Mi2a SRP	+							Idiopathic pulmonary fibrosis	Nintedanib	Remission/stable
20	32F	–	Ro52			+			+			Peripheral SpA	Certilizumab	Remission/stable
21	78F	–		SAE1								Autoimmune hepatitis		Remission/stable
22	33F	–	PmScl 100/75				+			+		Livedo-reticularis and previous peteacheal vasculitis rash in LL		Remission/stable
23	53F	+ H	Ro52		+				+			Diffuse systemic sclerosis	Steroid + MMF + Rituximab + Nintendinib	Remission/stable
24	62F	–		Mi2a								Pontine stroke and under workup for MS	Clopidogrel	Remission/stable
25	72M	–		Mi2b								AML and organizing pneumonia	Chemo + steroid taper for OP	Remission/stable
26	89M	–		Mi2a	+							UIP-ILD / IPF		Remission/stable
27	60F	–		Mi2b SAE1 SRP	+							IPF query RA related		Died
28	64F	–	Ro52				+			+		Discoid lupus	Was on steroid, HCQ + MMF	Remission/stable
29	60F	–	Ro52	MDA5	+							IPF query RA related	o2	Remission/stable
30	69M	–		Mi2b								COPD and asthma	Inhalers + on/off steroid	Remission/stable
31	76M	–	Ku									Hospital Acquired Pneumonia with parapneumonic effusions		Remission/stable
32	64M	–		PL-12	+							IPF		Remission/stable
33	79M	–		SAE1/PL-7								IPF	Nintedanib	Remission/stable
34	67M	–		NXP2	+							IPAF ILD secondary to CTD	Steroid +	Remission/stable
35	46F	+ S	U1snRNP Ro52							+		MCTD	HCQ	Remission/stable

*S* speckled, *H* homogenous, *C* cytoplasmic

### Clinical features

Tables 1 and 2 summarize the clinical features, ANA results, medication, and outcome of included cases. A creatine kinase (CK) level was performed in 52% of patients, with a median result of 69 (IQR 44.5–277, *p* = 0.57). Respiratory medicine accounted for the highest number of test requests (33%, 29/87), followed by rheumatology and immunology (24%, 21/87 each).

### Strong-positive MSA/MAA

Anti-PL12 was the most frequent strong positive MSA and anti-Ro52 the most common strong positive MAA (Table 3). The most frequently observed clinical features were arthralgia in 38% (20/52), ILD in 35% (18/52), and cutaneous manifestations in 29% (15/52). Arthritis was seen in 15% (8/52), Raynaud’s phenomenon in 15% (8/52), myositis in 13% (7/52), and malignancy in 12% (6/52). Thirteen percent (8/52) were diagnosed with dermatomyositis and 8% (4/52) with anti-synthetase syndrome.

**Table3:** The results of the antibodies for both positive and weakly positive

Antibody	Positive	Weakly positive
MSA		
Anti-PL-12	4	2
Anti-SAE1	3	3
Anti-Mi2	3	12
Anti-NXP2	2	1
Anti-Jo	2	1
Anti-SRP	2	5
Anti-PL7	2	2
Anti-EJ	2	1
Anti-OJ	2	1
Anti-MDA5	1	2
MAA		
Anti-Ro52	29	10
Anti-PMScl	7	5
Anti-Ku	3	–
Anti-U1RNP	2	2

### Weak-positive MSA/MAA

Anti-Mi2 was the most frequent weak-positive MSA and anti-Ro52 the most frequent weak-positive MAA (Table 3). The most common clinical manifestations were ILD in 34% (12/35), cutaneous manifestations in 20% (7/35), and arthralgia in 17% (6/35), with Raynaud’s phenomenon and arthritis in 11% each (4/35) and myositis and malignancy in 3% (1/35) each. No patients were diagnosed with IIM or anti-synthetase syndrome.

### Clinical correlates of positive MSA/MAA

A statistically significant association between arthralgia and a positive myositis panel was identified (*p* = 0.033) (Table 4). There were numerical differences for presentations of ILD (*p* = 0.975), myositis (*p* = 0.093), and cutaneous (*p* = 0.140) manifestations, but these did not reach statistical significance. A diagnosis of IIM was associated with a strong positive panel (*p* = 0.008). Symptom duration < 1 year was associated with a weakly positive panel (*p* = 0.022).

**Table 4:** Chi-square analysis between weak-positive and positive myositis panel

	Type				*p* value
	Weak-positive myositis panel		Positive myositis panel		
	Count	Column *N* %	Count	Column *N* %	
ILD	12	34.3	18	34.6	0.975
Arthritis	4	11.4	8	15.4	0.600
Arthralgia	6	17.1	20	38.5	0.033*
Myositis	1	2.9	7	13.5	0.093
Raynaud	4	11.4	8	15.4	0.600
Cutaneous	7	20.0	18	34.6	0.140
Malignancy	1	2.9	6	11.5	0.144
Final diagnosis					
Inflammatory myositis	0	0.0	8	15.4	0.008*
Interstitial lung disease	12	34.3	18	34.6	
Connective tissue disease	5	14.3	14	26.9	
Others	18	51.4	12	23.1	
Management					
Corticosteroid	3	8.6	5	9.6	0.115
Corticosteroid + immunosuppression	7	20.0	17	32.7	
Immunosuppression	3	8.6	12	23.1	
No treatment	11	31.4	9	17.3	
Others	11	31.4	9	17.3	
Outcome					
Died	2	5.7	3	5.8	0.773
Remission/stable	32	91.4	45	86.5	
Worsening	0	0.0	1	1.9	
Lost follow-up	1	2.9	3	5.8	
Duration					
= < 1 year	23	65.7	22	42.3	0.022*
2 years	6	17.1	14	26.9	
3 years	6	17.1	5	9.6	
4 years	0	0.0	8	15.4	
5 years	0	0.0	3	5.8	

*p < 0.05

Details of clinical features and diagnosis by individual MSA and MAA are shown in Supplementary Tables 1–7. There was no evident difference between single MSA/MAA positivity and positivity for more than one MSA/MAA and clinical features or diagnosis.

## Discussion

Our study shows that those with a strong positive myositis panel were more likely to be diagnosed with an IIM and were more likely to present with arthralgia. There were no diagnoses of IIM in the weakly positive myositis panel group.

A review of the literature shows variations of clinical presentation and serology across different populations. It is felt that genetic factors and environmental triggers may be responsible for this disparity [14]. For example, a study of a Greek population found that the most frequently detected MAA was anti-Ro-52 (30%), while the most frequently detected MSA was anti-Jo-1 (22%) [15]. In our total population, only 3% tested positive for anti-Jo-1.

Our study shows the association of MSA and MAA with IIM, ILD, and CTD are much higher at the strong positive antibody level when compared with the weak positive. However, the diagnostic yield of MSA was generally lower than previously reported studies [16, 17]. This may be because of a relatively short follow-up in our population compared to other published studies or may be due to testing in patients with a lower pre-test probability.

The American thoracic society/European respiratory society/Japanese respiratory society/Latin American thoracic society diagnostic guidelines recommend serial antibody testing in ILD to identify seroconversion and differentiate idiopathic pulmonary fibrosis (IPF) from CTD-ILD. In our study, 34% of all patients were diagnosed with ILD and respiratory having the highest number of requests. This shows the value of MSA testing in ILD as it may present with no or minimal symptoms suggestive of CTD [18]. As CTD- ILD confers a better prognosis and different treatment approach than IPF, it is of paramount importance to detect this subset at an early stage [19].

In our study, MSA were detected in many other inflammatory and non-inflammatory diseases. This finding is in contrast to the majority of prior studies. For instance, Vulseteke et al. reported positive MSA in half of patients with IIM compared to only 3.5% of patients with systemic inflammatory diseases and none in healthy controls [20]. This could suggest that MSA sensitivity and specificity vary from one testing lab to another [15, 16]. It may also be the case that there are differences in the populations being tested, with resultant variation in the pre-test probability.

We perform ANA in conjunction with the myositis panel to improve diagnostic performance [13]. 83% of weakly positive myositis panels in our cohort were ANA negative compared to 46% of strong positive panels (~ 93% correctly matched the non-ANA staining in the positive panel). A false-positive test should be considered if the autoantibody staining/pattern does not correlate with the ANA result and clinical context [9]. However, some MSA exhibit negative ANA testing due to cytoplasmic localisation, and as such negative ANA does not necessarily imply autoantibody negativity in IIM.

**Fig. 1:**
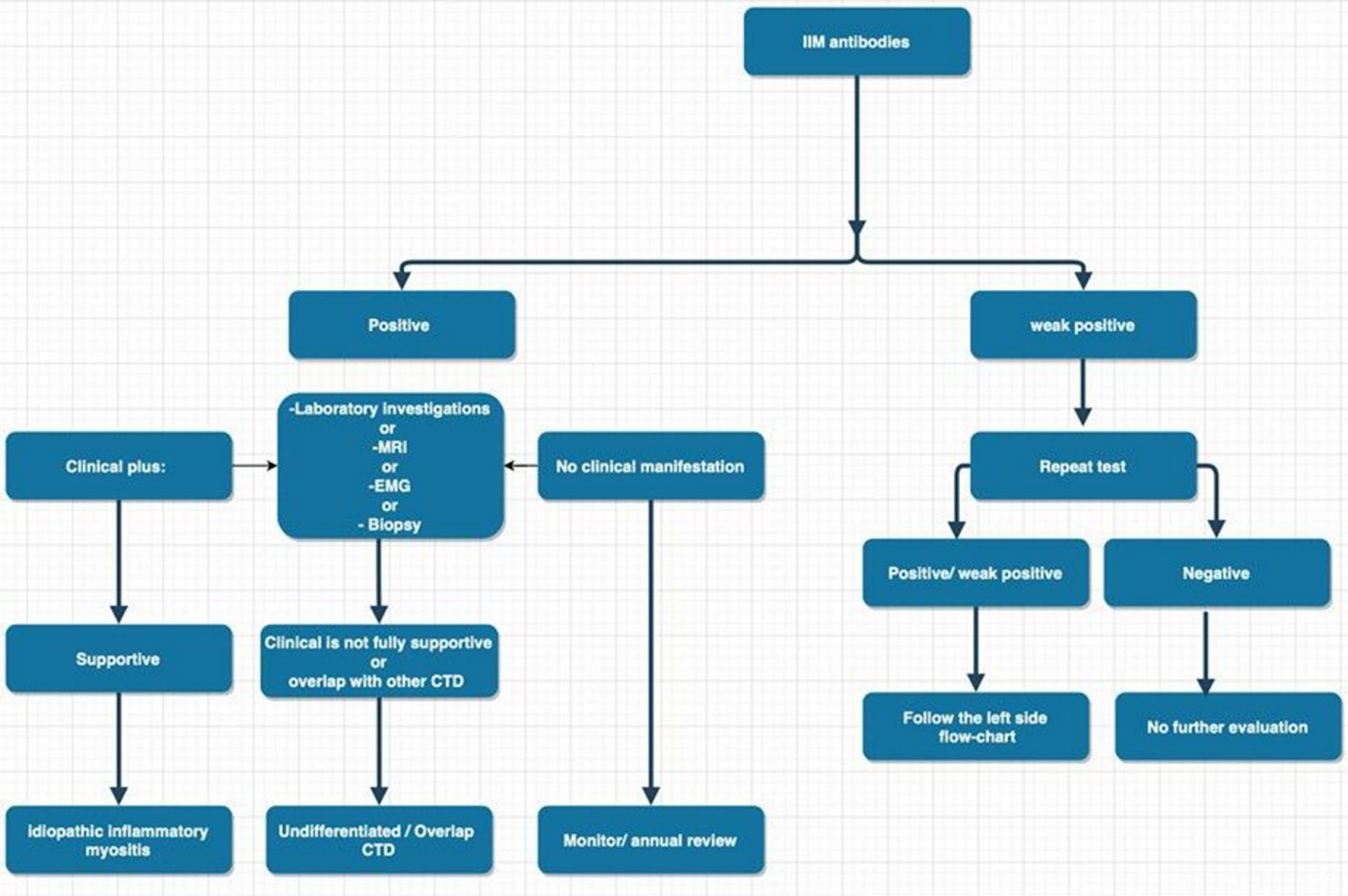
A proposed algorithm to guide interpretation of myositis antibody panel results

This study was not without its limitations. Our power to detect significant differences was impacted by a relatively small sample size and low number of IIM diagnoses. This highlights the need for larger collaborative studies to evaluate these rare conditions. This was a single-center study and our findings require confirmation in other settings to confirm external validity. Given the significant mortality and morbidity burden of IIM, early and accurate diagnosis should be a primary goal in all cases. Based on the above, we have proposed an algorithm to guide the interpretation of myositis antibody panel results, Fig. 1. This highlights our findings and suggests that weak-positive panels should be repeated to confirm the result.

The current EULAR/ACR guidelines suggest that clinical assessment and biopsy are the core components of the diagnostic approach to IIM. Our expanding knowledge of the importance of MSA/MAA suggests a key adjunctive role in diagnosis. Our study found that positive panels are more likely to be associated with IIM; however, a significant number of cases had no clinical features suggestive of CTD or IIM. A combined clinical and serological framework may be useful in IIM diagnosis.

## Supplementary Information

Below is the link to the electronic supplementary material.

Supplementary file1 (PDF 226 kb)

## Data Availability

Available from authors on request.
