# Supplementary material for: Association of extended myositis panel results, clinical features, and diagnoses: a single-center retrospective observational study
Source: Rheumatol Int. 2021 Oct 4;41(12):2133–46. doi: 10.1007/s00296-021-05012-0 (PMC8550373; doi:10.1007/s00296-021-05012-0)
Supplement: Supplementary file 1 — Supplementary file1 (PDF 226 kb) [file 296_2021_5012_MOESM1_ESM.pdf]

	Mi2b (N=3)	MDA5 (N=1)	NXP2 (N=2)	SAE1 (N=1)	Jo-1 (N=2)	SRP (N=2)	PL-7 (N=2)	PL-12 (N=4)	EJ (N=2)	OJ (N=2)	Total (N=21)
ILD											
**No**	3.0 (100.0%)	1.0 (100.0%)	2.0 (100.0%)	1.0 (100.0%)	0.0 (0.0%)	1.0 (50.0%)	0.0 (0.0%)	1.0 (25.0%)	1.0 (50.0%)	1.0 (50.0%)	11.0 (52.4%)
**Yes**	0.0 (0.0%)	0.0 (0.0%)	0.0 (0.0%)	0.0 (0.0%)	2.0 (100.0%)	1.0 (50.0%)	2.0 (100.0%)	3.0 (75.0%)	1.0 (50.0%)	1.0 (50.0%)	10.0 (47.6%)
Arthritis											
**No**	3.0 (100.0%)	1.0 (100.0%)	2.0 (100.0%)	1.0 (100.0%)	2.0 (100.0%)	2.0 (100.0%)	1.0 (50.0%)	3.0 (75.0%)	2.0 (100.0%)	1.0 (50.0%)	18.0 (85.7%)
**Yes**	0.0 (0.0%)	0.0 (0.0%)	0.0 (0.0%)	0.0 (0.0%)	0.0 (0.0%)	0.0 (0.0%)	1.0 (50.0%)	1.0 (25.0%)	0.0 (0.0%)	1.0 (50.0%)	3.0 (14.3%)
Arthralgia											
**No**	2.0 (66.7%)	0.0 (0.0%)	1.0 (50.0%)	1.0 (100.0%)	2.0 (100.0%)	2.0 (100.0%)	1.0 (50.0%)	3.0 (75.0%)	1.0 (50.0%)	1.0 (50.0%)	14.0 (66.7%)
**Yes**	1.0 (33.3%)	1.0 (100.0%)	1.0 (50.0%)	0.0 (0.0%)	0.0 (0.0%)	0.0 (0.0%)	1.0 (50.0%)	1.0 (25.0%)	1.0 (50.0%)	1.0 (50.0%)	7.0 (33.3%)
myositis											
**No**	3.0 (100.0%)	1.0 (100.0%)	1.0 (50.0%)	0.0 (0.0%)	2.0 (100.0%)	2.0 (100.0%)	1.0 (50.0%)	4.0 (100.0%)	2.0 (100.0%)	1.0 (50.0%)	17.0 (81.0%)
**Yes**	0.0 (0.0%)	0.0 (0.0%)	1.0 (50.0%)	1.0 (100.0%)	0.0 (0.0%)	0.0 (0.0%)	1.0 (50.0%)	0.0 (0.0%)	0.0 (0.0%)	1.0 (50.0%)	4.0 (19.0%)
Raynaud											
**No**	3.0 (100.0%)	1.0 (100.0%)	2.0 (100.0%)	1.0 (100.0%)	1.0 (50.0%)	1.0 (50.0%)	1.0 (50.0%)	4.0 (100.0%)	2.0 (100.0%)	2.0 (100.0%)	18.0 (85.7%)
**Yes**	0.0 (0.0%)	0.0 (0.0%)	0.0 (0.0%)	0.0 (0.0%)	1.0 (50.0%)	1.0 (50.0%)	1.0 (50.0%)	0.0 (0.0%)	0.0 (0.0%)	0.0 (0.0%)	3.0 (14.3%)
cutaneous											
**No**	2.0 (66.7%)	0.0 (0.0%)	1.0 (50.0%)	0.0 (0.0%)	2.0 (100.0%)	1.0 (50.0%)	0.0 (0.0%)	3.0 (75.0%)	2.0 (100.0%)	1.0 (50.0%)	12.0 (57.1%)
**Yes**	1.0 (33.3%)	1.0 (100.0%)	1.0 (50.0%)	1.0 (100.0%)	0.0 (0.0%)	1.0 (50.0%)	2.0 (100.0%)	1.0 (25.0%)	0.0 (0.0%)	1.0 (50.0%)	9.0 (42.9%)
malignancy											
**No**	3.0 (100.0%)	1.0 (100.0%)	2.0 (100.0%)	1.0 (100.0%)	2.0 (100.0%)	1.0 (50.0%)	2.0 (100.0%)	3.0 (75.0%)	2.0 (100.0%)	2.0 (100.0%)	19.0 (90.5%)
**Yes**	0.0 (0.0%)	0.0 (0.0%)	0.0 (0.0%)	0.0 (0.0%)	0.0 (0.0%)	1.0 (50.0%)	0.0 (0.0%)	1.0 (25.0%)	0.0 (0.0%)	0.0 (0.0%)	2.0 (9.5%)

Supplementary table 1: clinical associations of strong-positive MSA

	PMScl (100/75) (N=4)	U1snRNP (N=2)	Ku (N=2)	Ro52 (N=28)	Total (N=36)
ILD					
**No**	3.0 (75.0%)	2.0 (100.0%)	2.0 (100.0%)	19.0 (67.9%)	26.0 (72.2%)
**Yes**	1.0 (25.0%)	0.0 (0.0%)	0.0 (0.0%)	9.0 (32.1%)	10.0 (27.8%)
Arthritis					
**No**	4.0 (100.0%)	0.0 (0.0%)	2.0 (100.0%)	24.0 (85.7%)	30.0 (83.3%)
**Yes**	0.0 (0.0%)	2.0 (100.0%)	0.0 (0.0%)	4.0 (14.3%)	6.0 (16.7%)
Arthralgia					
**No**	4.0 (100.0%)	0.0 (0.0%)	0.0 (0.0%)	15.0 (53.6%)	19.0 (52.8%)
**Yes**	0.0 (0.0%)	2.0 (100.0%)	2.0 (100.0%)	13.0 (46.4%)	17.0 (47.2%)
myositis					
**No**	3.0 (75.0%)	1.0 (50.0%)	2.0 (100.0%)	26.0 (92.9%)	32.0 (88.9%)
**Yes**	1.0 (25.0%)	1.0 (50.0%)	0.0 (0.0%)	2.0 (7.1%)	4.0 (11.1%)
Raynaud					
**No**	3.0 (75.0%)	1.0 (50.0%)	2.0 (100.0%)	24.0 (85.7%)	30.0 (83.3%)
**Yes**	1.0 (25.0%)	1.0 (50.0%)	0.0 (0.0%)	4.0 (14.3%)	6.0 (16.7%)
cutaneous					
**No**	2.0 (50.0%)	1.0 (50.0%)	2.0 (100.0%)	20.0 (71.4%)	25.0 (69.4%)
**Yes**	2.0 (50.0%)	1.0 (50.0%)	0.0 (0.0%)	8.0 (28.6%)	11.0 (30.6%)
malignancy					
**No**	4.0 (100.0%)	2.0 (100.0%)	2.0 (100.0%)	24.0 (85.7%)	32.0 (88.9%)
**Yes**	0.0 (0.0%)	0.0 (0.0%)	0.0 (0.0%)	4.0 (14.3%)	4.0 (11.1%)

Supplementary table 2: clinical associations of strong-positive MAA

	Mi2a (N=3)	Mi2b (N=6)	TIF1 (N=1)	MDA5 (N=1)	NXP2 (N=1)	SAE1 (N=3)	SRP (N=3)	PL-7 (N=2)	PL-12 (N=2)	OJ (N=1)	Total (N=23)
ILD											
**No**	2.0 (66.7%)	4.0 (66.7%)	1.0 (100.0%)	0.0 (0.0%)	0.0 (0.0%)	2.0 (66.7%)	0.0 (0.0%)	2.0 (100.0%)	1.0 (50.0%)	1.0 (100.0%)	13.0 (56.5%)
**Yes**	1.0 (33.3%)	2.0 (33.3%)	0.0 (0.0%)	1.0 (100.0%)	1.0 (100.0%)	1.0 (33.3%)	3.0 (100.0%)	0.0 (0.0%)	1.0 (50.0%)	0.0 (0.0%)	10.0 (43.5%)
Arthritis											
**No**	2.0 (66.7%)	6.0 (100.0%)	1.0 (100.0%)	1.0 (100.0%)	1.0 (100.0%)	3.0 (100.0%)	3.0 (100.0%)	1.0 (50.0%)	2.0 (100.0%)	0.0 (0.0%)	20.0 (87.0%)
**Yes**	1.0 (33.3%)	0.0 (0.0%)	0.0 (0.0%)	0.0 (0.0%)	0.0 (0.0%)	0.0 (0.0%)	0.0 (0.0%)	1.0 (50.0%)	0.0 (0.0%)	1.0 (100.0%)	3.0 (13.0%)
Arthralgia											
**No**	2.0 (66.7%)	6.0 (100.0%)	1.0 (100.0%)	1.0 (100.0%)	1.0 (100.0%)	3.0 (100.0%)	3.0 (100.0%)	2.0 (100.0%)	2.0 (100.0%)	0.0 (0.0%)	21.0 (91.3%)
**Yes**	1.0 (33.3%)	0.0 (0.0%)	0.0 (0.0%)	0.0 (0.0%)	0.0 (0.0%)	0.0 (0.0%)	0.0 (0.0%)	0.0 (0.0%)	0.0 (0.0%)	1.0 (100.0%)	2.0 (8.7%)
myositis											
**No**	3.0 (100.0%)	6.0 (100.0%)	1.0 (100.0%)	1.0 (100.0%)	1.0 (100.0%)	3.0 (100.0%)	3.0 (100.0%)	2.0 (100.0%)	1.0 (50.0%)	1.0 (100.0%)	22.0 (95.7%)
**Yes**	0.0 (0.0%)	0.0 (0.0%)	0.0 (0.0%)	0.0 (0.0%)	0.0 (0.0%)	0.0 (0.0%)	0.0 (0.0%)	0.0 (0.0%)	1.0 (50.0%)	0.0 (0.0%)	1.0 (4.3%)
Raynaud											
**No**	3.0 (100.0%)	6.0 (100.0%)	1.0 (100.0%)	1.0 (100.0%)	1.0 (100.0%)	3.0 (100.0%)	3.0 (100.0%)	2.0 (100.0%)	2.0 (100.0%)	1.0 (100.0%)	23.0 (100.0%)
**Yes**	0.0 (0.0%)	0.0 (0.0%)	0.0 (0.0%)	0.0 (0.0%)	0.0 (0.0%)	0.0 (0.0%)	0.0 (0.0%)	0.0 (0.0%)	0.0 (0.0%)	0.0 (0.0%)	0.0 (0.0%)
cutaneous											
**No**	2.0 (66.7%)	5.0 (83.3%)	1.0 (100.0%)	1.0 (100.0%)	1.0 (100.0%)	3.0 (100.0%)	3.0 (100.0%)	1.0 (50.0%)	2.0 (100.0%)	1.0 (100.0%)	20.0 (87.0%)
**Yes**	1.0 (33.3%)	1.0 (16.7%)	0.0 (0.0%)	0.0 (0.0%)	0.0 (0.0%)	0.0 (0.0%)	0.0 (0.0%)	1.0 (50.0%)	0.0 (0.0%)	0.0 (0.0%)	3.0 (13.0%)
malignancy											
**No**	3.0 (100.0%)	6.0 (100.0%)	1.0 (100.0%)	1.0 (100.0%)	1.0 (100.0%)	3.0 (100.0%)	3.0 (100.0%)	2.0 (100.0%)	2.0 (100.0%)	1.0 (100.0%)	23.0 (100.0%)
**Yes**	0.0 (0.0%)	0.0 (0.0%)	0.0 (0.0%)	0.0 (0.0%)	0.0 (0.0%)	0.0 (0.0%)	0.0 (0.0%)	0.0 (0.0%)	0.0 (0.0%)	0.0 (0.0%)	0.0 (0.0%)

Supplementary table 3: clinical associations of weak-positive MSA

	PMScl(100/75) (N=4)	U1snRNP (N=1)	Ku (N=1)	Ro52 (N=10)	Total (N=16)
ILD					
**No**	4.0 (100.0%)	1.0 (100.0%)	1.0 (100.0%)	7.0 (70.0%)	13.0 (81.2%)
**Yes**	0.0 (0.0%)	0.0 (0.0%)	0.0 (0.0%)	3.0 (30.0%)	3.0 (18.8%)
Arthritis					
**No**	3.0 (75.0%)	1.0 (100.0%)	1.0 (100.0%)	8.0 (80.0%)	13.0 (81.2%)
**Yes**	1.0 (25.0%)	0.0 (0.0%)	0.0 (0.0%)	2.0 (20.0%)	3.0 (18.8%)
Arthralgia					
**No**	1.0 (25.0%)	1.0 (100.0%)	1.0 (100.0%)	8.0 (80.0%)	11.0 (68.8%)
**Yes**	3.0 (75.0%)	0.0 (0.0%)	0.0 (0.0%)	2.0 (20.0%)	5.0 (31.2%)
myositis					
**No**	4.0 (100.0%)	0.0 (0.0%)	1.0 (100.0%)	10.0 (100.0%)	15.0 (93.8%)
**Yes**	0.0 (0.0%)	1.0 (100.0%)	0.0 (0.0%)	0.0 (0.0%)	1.0 (6.2%)
Raynaud					
**No**	3.0 (75.0%)	1.0 (100.0%)	1.0 (100.0%)	7.0 (70.0%)	12.0 (75.0%)
**Yes**	1.0 (25.0%)	0.0 (0.0%)	0.0 (0.0%)	3.0 (30.0%)	4.0 (25.0%)
cutaneous					
**No**	3.0 (75.0%)	1.0 (100.0%)	1.0 (100.0%)	6.0 (60.0%)	11.0 (68.8%)
**Yes**	1.0 (25.0%)	0.0 (0.0%)	0.0 (0.0%)	4.0 (40.0%)	5.0 (31.2%)
malignancy					
**No**	4.0 (100.0%)	1.0 (100.0%)	1.0 (100.0%)	9.0 (90.0%)	15.0 (93.8%)
**Yes**	0.0 (0.0%)	0.0 (0.0%)	0.0 (0.0%)	1.0 (10.0%)	1.0 (6.2%)

Supplementary table 4: clinical associations of weak-positive MAA

	Mi2b (N=3)	MDA5 (N=1)	NXP2 (N=2)	SAE1 (N=1)	Jo-1 (N=2)	SRP (N=2)	PL-7 (N=2)	PL-12 (N=4)	EJ (N=2)	OJ (N=2)	Total (N=21)
**Final diagnosis**											
**Inflammatory myositis**	1.0 (33.3%)	1.0 (100.0%)	1.0 (50.0%)	1.0 (100.0%)	0.0 (0.0%)	0.0 (0.0%)	0.0 (0.0%)	0.0 (0.0%)	0.0 (0.0%)	0.0 (0.0%)	4.0 (19.0%)
**Interstitial lung disease**	0.0 (0.0%)	0.0 (0.0%)	0.0 (0.0%)	0.0 (0.0%)	2.0 (100.0%)	1.0 (50.0%)	2.0 (100.0%)	3.0 (75.0%)	1.0 (50.0%)	1.0 (50.0%)	10.0 (47.6%)
**Connective tissue disease**	0.0 (0.0%)	0.0 (0.0%)	0.0 (0.0%)	0.0 (0.0%)	0.0 (0.0%)	1.0 (50.0%)	0.0 (0.0%)	0.0 (0.0%)	1.0 (50.0%)	1.0 (50.0%)	3.0 (14.3%)
**Others**	2.0 (66.7%)	0.0 (0.0%)	1.0 (50.0%)	0.0 (0.0%)	0.0 (0.0%)	0.0 (0.0%)	0.0 (0.0%)	1.0 (25.0%)	0.0 (0.0%)	0.0 (0.0%)	4.0 (19.0%)

Supplementary table 5: strong-positive MSA and final diagnosis

	PMScl (100/75) (N=4)	U1snRNP (N=2)	Ku (N=2)	Ro52 (N=28)	Total (N=36)
**Final diagnosis**					
**Inflammatory myositis**	1.0 (25.0%)	0.0 (0.0%)	0.0 (0.0%)	3.0 (10.7%)	4.0 (11.1%)
**Interstitial lung disease**	1.0 (25.0%)	0.0 (0.0%)	0.0 (0.0%)	9.0 (32.1%)	10.0 (27.8%)
**Connective tissue disease**	1.0 (25.0%)	2.0 (100.0%)	0.0 (0.0%)	9.0 (32.1%)	12.0 (33.3%)
**Others**	1.0 (25.0%)	0.0 (0.0%)	2.0 (100.0%)	7.0 (25.0%)	10.0 (27.8%)

Supplementary table 6: strong-positive MAA and final diagnosis

	Mi2a (N=3)	Mi2b (N=6)	TIF-1 (N=1)	MDA5 (N=1)	NXP2 (N=1)	SAE1 (N=3)	SRP (N=3)	PL-7 (N=2)	PL-12 (N=2)	OJ (N=1)	Total (N=23)
**Final diagnosis**											
**Interstitial lung disease**	1.0 (33.3%)	2.0 (33.3%)	0.0 (0.0%)	1.0 (100.0%)	1.0 (100.0%)	1.0 (33.3%)	3.0 (100.0%)	1.0 (50.0%)	1.0 (50.0%)	0.0 (0.0%)	11.0 (47.8%)
**Connective tissue disease**	1.0 (33.3%)	0.0 (0.0%)	0.0 (0.0%)	0.0 (0.0%)	0.0 (0.0%)	0.0 (0.0%)	0.0 (0.0%)	1.0 (50.0%)	0.0 (0.0%)	0.0 (0.0%)	2.0 (8.7%)
**Others**	1.0 (33.3%)	4.0 (66.7%)	1.0 (100.0%)	0.0 (0.0%)	0.0 (0.0%)	2.0 (66.7%)	0.0 (0.0%)	0.0 (0.0%)	1.0 (50.0%)	1.0 (100.0%)	10.0 (43.5%)

Supplementary table 7: weak-positive MSA and final diagnosis

	PMScl (100/75) (N=4)	U1snRNP (N=1)	Ku (N=1)	Ro52 (N=10)	Total (N=16)
**Final diagnosis**					
**Interstitial lung disease**	0.0 (0.0%)	0.0 (0.0%)	0.0 (0.0%)	2.0 (20.0%)	2.0 (12.5%)
**Connective tissue disease**	0.0 (0.0%)	0.0 (0.0%)	0.0 (0.0%)	4.0 (40.0%)	4.0 (25.0%)
**Others**	4.0 (100.0%)	1.0 (100.0%)	1.0 (100.0%)	4.0 (40.0%)	10.0 (62.5%)

Supplementary table 8: weak-positive MAA and final diagnosis
